# Bad rules rather than slow discretion? A critical appraisal of EU stabilisation policy

**DOI:** 10.1007/s42495-021-00063-4

**Published:** 2021-06-11

**Authors:** Bernd Lucke, Bodo R. Neumann

**Affiliations:** Department of Economics, Universität Hamburg, Hamburg, Germany

**Keywords:** Fiscal union, Stabilisation function, Eurozone budget, Cohesion policy, E62, H77

## Abstract

The EU reacted swiftly to the economic dimension of Covid-19 by designing new instruments to support the fiscal policy of Member States. But entry into force and implementation was slow due to various political hurdles with little action taking effect by the end of 2020. In a draft law currently under consideration in the European Parliament, the Commission proposes speedier crisis responses using a rules-based approach. We analyse the legal and economic aspects of this so-called “European Investment Stabilisation Function” (EISF) and argue that a rules-based policy may be inefficient and detrimental to important EU policy objectives. For instance, in the Covid-19 crisis, most of the EISF funds would have supported only the wealthiest Member States. In general, we show that well-intended EU-funded stabilisation measures may actually be counterproductive in terms of EU cohesion, suboptimal in terms of stabilisation and regressive in terms of cross-country income distribution.

## Introduction

The Covid-19 pandemic has caused a major recession for all European economies, often unprecedented in size in postwar or post-communist times. There is wide consensus among economists that debt-financed expansionary fiscal policy is the best policy response to the economic dimension of this crisis. But many European countries – particular countries of the Eurozone – had high levels of debt already prior to Covid-19. Despite historically low interest rates and massive purchases of government bonds through the European Central Bank’s Pandemic Emergency Purchase Programme (PEPP), the resurgence of the European sovereign debt crisis is clearly a concern among policy makers and economists.

EU leaders have reacted to this challenge by a number of improvised measures, not envisaged prior to the outbreak of the crisis. Among these is a 100 billion € support programme to mitigate unemployment risks in an emergency (SURE), a 240 billion € credit line for Member States of the European Stability Mechanism (ESM), and a 750 billion € recovery instrument “Next Generation EU” intended to supply debt-financed grants and credits to EU Member States via the EU budget.

These measures have in common that they make funds available without requiring Member States to increase their issuance of government bonds. Rather, the EU or the ESM will issue sovereign bonds on own account. Since this debt is backed either proportionately or mutually by all Member States, the countries hardest hit by the crisis will–so the thinking goes–have greater access to emergency funds than under a stand-alone scenario.

However, the financial instruments have been slow to take effect. At the time of writing (December 2020) no ESM country had applied to use the 240 billion € credit line made available via the ESM. Rumour has it that Member States shy away from a perceived stigma of being the recipient of ESM credits, which, according to Articles 3 and 12 of the ESM Treaty, are granted under “strict conditionality” only. Moreover, while the European Council agreed in May 2020 on the design of the 750 billion € recovery instrument, it took EU leaders until December 2020 to clear political hurdles related to the “rule-of-law” mechanism and “Next Generation EU” is still waiting to see entry into force while the second wave of Covid-19 plagues the Continent. Finally, from the 100 billion € SURE programme, approved in May 2020, no disbursement took place before end-October and the actual instalments paid at the time of writing were only 17 billion €.

While there is no doubt that European leaders aimed at making available large European funds almost immediately after the size of the economic crisis became known, not much speedy action in response to Covid-19 can be witnessed.

This raises the question if other options to respond to a major crisis are available – and, if they are better suited to serve the Union’s overarching goals of economic growth and cohesion. One option relies on the idea that financial support for countries hit by a major shock may be “automatically set on the basis of a formula” enshrined in European legislation, cf. European Commission (2018, p. 12).

This option was brought forward by the Commission as a legislative proposal for the establishment of a “European Investment Stabilisation Function” (EISF).^1^ Such a proposal is – since May 2018—under consideration by the European Parliament and the Council.^2^ It is supposed to ensure funds for macroeconomic stabilisation in case of asymmetric shocks in a “swift and lean decision-making procedure”, cf. European Commission (2018, p. 4), by foreseeing simple mathematical rules to determine eligibility and size of financial aid for Member states.

Rules rather than discretion? This is indeed the question, but under a different angle than in Kydland and Prescott’s [13] classical paper: For in terms of crisis response, European policy makers hardly bother about *time consistency*. Rather, the trade-off is *time delays* linked to discretionary decision making versus the *consistency* of rules-based support measures with the general economic policy objectives of the Union.

We will, in the sequel, shed some light on the rules-based approach advocated by the Commission in its EISF proposal, and argue that support measures based on pre-agreed formulae may allow at best marginally faster crisis responses, but do so only at considerable costs.

We build on and extend results by Lucke and Neumann [14] by showing that the EISF rules would have led to grave misallocations of funds during Covid-19 and previous crises, suboptimal effects in terms of stabilisation policy and would have been distributionally regressive. Even worse, both discretionary support measures and rules-based allocation of emergency funds create disincentives for Member States to counter the crisis swiftly with own resources and may induce strategical delay.

Given the serious weaknesses of rules-based and discretionary approaches to crises response at Union level, we argue that it remains indispensable for Member States to preserve (or create) own fiscal space in normal times. Automatic stabilizers and fiscal stimuli at national level are well-tested means of stabilisation policy. Union measures, by contrast, face design and implementation issues which make them unreliable if not undesirable.

The paper is organized as follows: Sect. “Macroeconomomic Stabilisation at EU level” reviews the political development towards fiscal capacity of the Union and relates it to the literature. Section “The Legal Framework” discusses the legal constraints of EU stabilisation policy. Sections “The European Investment Stabilisation Function” and “Economic Analysis of the Commission Proposal present and analyse the Commission’s EISF proposal. Section “EISF and Covid-19” studies how the EISF would have worked out during the Covid-19 crisis. In Sect. “Historic Simulation and Disincentive Effects” we present historical simulations for previous economic crises in the EU and discuss disincentive effects. We conclude in Sect. “Conclusions”.

## Macroeconomomic stabilisation at EU level

By introducing the Euro 20 years ago, the European Union (EU) opted for a common monetary policy while Member States would continue to have sovereignty over their fiscal policy. This design has received increased criticism, especially since the beginning of the sovereign debt crisis in 2010. For several years, researchers and academic policy advisors have called for more fiscal policy coordination and centralized decision making (e.g. Wolff [19], Allard et al. [1]). Some economists claim that a common currency *requires* a common fiscal policy (cf. Glienicker Gruppe [8], Farhi and Werning [6].

For some time, these positions were highly controversial. For instance, Galí and Monacelli (2008) show that a monetary union with decentral fiscal policies may well achieve the optimum outcome for the Union as a whole. Matthes et al. [15] also argue that existing instruments are sufficient and a further fiscal integration in the EU is unnecessary. Kehoe and Pastorino [12] argue that fiscal risk sharing is not necessary in a monetary union if developed financial markets are in place. Also, the influential German Council of Economic Experts in its annual report [7] speaks out against a fiscal capacity at Union level.

Nevertheless, considerations already developed by the former European Economic Union in the Marjolin Report Commission of the European Communities [3] and especially by Mc Dougall [16] were taken up again at highest level in 2015. In the “Five Presidents’ Report”, the EU aimed at “completing Europe’s Economic and Monetary Union” (cf. European Commission [5]). The Commission called for setting up “a macroeconomic stabilisation function for the Euro area” (in the following “stabilisation function”) to create a fiscal union until 2025 at the latest.

Similarly, Commission President Juncker addressed the presidents of the co-legislators on this issue in a Letter of Intent appended to his State of the Union address on 13.09.2017 Juncker and Timmermans [11]. He announced proposals for the “creation of a dedicated Euro area budget line within the EU budget” that shall provide for a “stabilisation function”.

However, an EU stabilisation function might face significant legal obstacles since “any decision to set up such an instrument would need to take due account of possible legal constraints.” (European Commission (2017a)). Undoubtedly, a solid legal basis for a macroeconomic stabilisation function does not exist in European primary law. Treaty change is an option, but currently unlikely. On the one hand, a significant number of the Member States seem to be unwilling to open the door for establishing a fiscal union in the first place Hansegroup [9]. On the other hand, even those willing to support further fiscal integration are concerned that the unanimity requirement for Treaty change gives undue leverage to some Member States pursuing unrelated – and possibly unwanted – political objectives.

Is there a way to implement a common fiscal policy without Treaty change? The Commission (in its EISF proposal) and the Council (in its proposal for a “Budgetary Instrument for Convergence and Competitiveness” (BICC)) seem to have identified the EU’s competence for cohesion policy (Article 175 paragraph 3 of the Treaty on the Functioning of the European Union (TFEU)) as the most promising avenue.^3^ This article serves as the legal basis in both proposals, cf. Council of the European Union (2019).

Cohesion policy and macroeconomic stabilisation differ significantly in various aspects. If Article 175 TFEU is used as the legal basis for the stabilisation function, the EU’s macroeconomic stabilisation policy must conform to the goals and standards set for cohesion policy. As we will show, this creates challenges that cannot easily be overcome and may induce disincentives and misallocations.

## The legal framework

The European Union derives its competences from the principle of conferral (Treaty on the European Union, Article 5). Thus, each legislative act of the Union requires an explicit legal basis in European primary legislation, i.e. either in the Treaty on the European Union (TEU) or in the Treaty on the Functioning of the European Union (TFEU). But Title VIII of the TFEU (on Economic and Monetary Policy) does not provide a legal basis for a stabilization function, which is why the Commission resorts to Article 175 TFEU in Title XVIII (on Cohesion).

As Horn [10] and < self-citation > have pointed out, this may be legally inadmissible, because cohesion policy is neither super- nor subordinate to economic and monetary policy. This was emphasized in 2009 by the European Court of Justice (ECJ) which ruled that Title XVIII provides the legal basis for policies whose “content does not extend beyond the scope of the Community’s policy on economic and social cohesion” ECJ (4, No. 46)).

In other words: The EU’s authorization to pursue cohesion policy cannot be re-dedicated to achieve other goals of the Union or of the Eurogroup (cf. Vaubel [18]. In a legal opinion on the EISF proposal, Horn [10] writes: „The political goal of economic, social and territorial cohesion is inapt to legitimize policy measures that are considered necessary to attain other Treaty objectives for which the necessary authorization is not provided in the respective other Titles of the Treaty.”

The EISF proposal pays tribute to this legal constraint by emphasizing (also in European Commission [7, 9]) that the EISF shall enhance the Eurozone’s resilience to “asymmetric shocks”. This wording has presumably been chosen because asymmetric shocks may undermine the cohesion of the Union and would thus justify policy measures based on Title XVIII TFEU.

From a stabilisation perspective, however, this design seems bizarre. An adverse shock hitting the Eurozone may well be symmetric, e. g. the financial crisis or the Covid-19 crisis. There is no economic reason why the proposal for a stabilisation function should be confined to *asymmetric* shocks. As we will show later, the Commission’s legal text essentially defines just any shock as “asymmetric”.

Further restrictions originate from the objectives of cohesion policy as stated in primary law. In Article 174 TFEU, these are defined to be *regional* and *structural*. Article 174 TFEU states that “the Union shall aim at reducing disparities between the levels of development of the various regions and the backwardness of the least favoured regions.” Among the regions concerned, particular attention shall be paid to “rural areas, areas affected by industrial transition, and regions which suffer from severe and permanent natural or demographic handicaps such as the northernmost regions with very low population density and island, crossborder and mountain regions.”

This distinguishes cohesion policy from stabilization policy which refers to *national* entities, i. e. Member States. A general investment subsidy at country level is therefore hardly an instrument of cohesion policy.

While an exhaustive presentation of the legal constraints concerning the EISF is beyond the scope of this paper, the practical restrictions resulting from the legal framework are highly relevant for economic policy. For this reason we now focus on the economic analysis of the Commission’s proposal.

## The european investment stabilisation function

In the Commission’s draft regulation for the establishment of a European Investment Stabilisation Function, Member States which belong to the Eurozone or participate in the Exchange Rate Mechanism II (ERM II), shall be granted financial assistance if they are hit by a severe asymmetric shock. Support shall take the form of loans for the funding of public investment projects and the form of grants, which cover 100% of the interest cost incurred on the loans. We call the latter the interest subsidy.^4^

An “asymmetric shock” is determined by a “double activation trigger”. Firstly, the quarterly national unemployment rate (in the following $u^{h}$) has to exceed “the average unemployment rate in the Member State concerned over a period of 60 quarters preceding the quarter during which the request is made”. Secondly, the same unemployment rate has to have “increased above one percentage point in comparison to the unemployment rate observed in the same quarter of the previous year” (in the following $u_{0}$).

The second component of the „double unemployment trigger “ essentially determines how high the granted financial support will be. Let $\delta : = u^{h} - \left( {u_{0} + 1} \right)$ be the difference between $u^{h}$ and the unemployment rate $u_{0}$ increased by one percentage point (both measured in percentage points). The following simple equation is employed by the EU to calculate the loan amount *S*:

1 $$S = \alpha \beta \delta I*$$

where $\alpha$ and $\beta$ are exogenous parameters. $I*$ is the fictitious level of public investment that the Member State concerned would invest if it invested the same share of its gross domestic product as the EU average.

In the Commission’s proposal, the parameters $\alpha$ and $\beta$ take the values $\alpha = 11.5$ and $\beta = 0.66$.^5^ One purpose of these values is to set an annual ceiling for the maximum amount of loans granted to a Member State, i. e. $S \le \alpha I*$. However, this ceiling is set quite high with $\alpha = 11.5$. Note that in 2019, public investment accounted for about 3% of the Union’s GDP. Thus, the ceiling for a potential loan is about a third of its GDP! Moreover, the Commission has discretion to increase $\beta$ from its basic value $\beta = 0.66$ to $\beta = 1$.

The Commission’s discretion is limited by the fact that at no point in time loans granted under the EISF shall exceed a maximum of 30 billion €. However, the ceiling only refers to support from the Union budget and explicitly allows the European Stabilisation Mechanism ESM (or its successor) to approve complementary loans on the same conditions. Since in a major crisis formula (1) implies loans which easily exceed 30 billion € even for single countries, the Commission probably aims at such complementary financing.

A Member State may request an EISF loan once a year and should it meet the elegibility and activation criteria, the Commission shall automatically calculate the amount of the loan on the basis of the formula described above (cf. Recital 24).

## Economic analysis of the commission proposal

In the next sections, we provide an economic analysis of the EISF proposal, both qualitatively (this section) and quantitatively (Sect. “EISF and Covid-19”). Qualitatively, we focus on the potential conflict between cohesion policy and macroeconomic stabilisation policy: Cohesion policy is commonly understood as improving the economic *structure* of disadvantaged regions—typically irrespective of cyclical economic fluctuations. It is mainly focussed on *supply*-side conditions and unfolds its effects only gradually over time.

By contrast, macroeconomic stabilisation policy involves economy-wide (rather than regional) measures which stimulate aggregate *demand* in the short-term and which are applied solely during crises and recessions.

Moreover, stabilisation policy differs from cohesion policy as it is oriented toward known, historically or geographically determined country-specific conditions. These "grown" conditions have nothing in common with an unexpected aggregate shock, typically coming from outside, as the trigger of stabilisation policy.

Cohesion policy and stabilisation policy are thus rather polar manifestations of national economic policy along several dimensions. The Commission tries to overcome this by anchoring the legislative proposal in the field of cohesion policy and simultaneously expanding the traditional understanding of cohesion policy. This is done in the “Common Provisions Regulation”, a separate legislative act^6^ which defines as eligible public investment any investment focussed on a “smarter Europe”, a “greener, low-carbon Europe”, a “more connected Europe”, a “more social Europe”, and a “Europe closer to citizens”. By this, almost any future stabilization policy measure could be classified as cohesion policy.

Moreover, the Commission widens the concept of "asymmetric shocks" to such an extent that it de facto includes symmetric shocks: Recital 13 defines “asymmetric shocks” as those which affect one or more Member States significantly more than the average of the Member States. Obviously, this will always be the case since it is inherent in the average that some countries are affected more severely than the average. Consequently, a shock that affects all countries adversely – but to varying degrees – would be an asymmetric rather than a symmetric shock. And indeed, the financial crisis of 2008 and 2009, which is based on an undoubtedly symmetric shock, is explicitly cited in Recital 4 of the EISF proposal as an example of an asymmetric shock.

It should also be mentioned that in the Commission’s proposal, the term “shock” is used in an unconventional way. According to the definition in Article 4, just *any* event that meets the double activation criterion is considered to be a “shock”. It is not specified that a shock must be an exogenous event beyond the control of the Member State concerned. Therefore, also an endogenous event, for which the Member State bears full responsibility, can be considered a shock in the sense of the EISF.

Hence, the EISF legislative proposal would empower the EU to grant interest-free loans in response to almost any adverse event which has a sufficiently strong impact on the unemployment of a (Eurozone!) Member State. But cohesion applies to the Union as a whole, not only to the Eurozone. Since Eurozone countries are on average richer than non-Eurozone countries, it is not obvious that financial support for Eurozone countries benefits the EU’s cohesion.

Moreover, the delimitation of cohesion and stabilization policy devised by the Commission may be highly problematic. On the one hand, stabilization may be suboptimal if the funds have to be spent in line with cohesion funds regulations. On the other hand, widening the criteria which define cohesion spending may be detrimental for cohesion policy since resources earmarked for cohesion may be channelled to other purposes, e. g. stabilization. Moreover, the necessity to react quickly to an unfolding crisis is likely to see traditional cohesion projects being approved as “stabilization” even if the projects are inferior or still insufficiently prepared. This could have a lasting negative impact on the efficiency of cohesion policy.

## EISF and Covid-19

To test the effectiveness of a stabilisation function designed as in the EISF proposal, we simulate it using recent data of the Covid-19 crisis. We analyse the size and timing by which each Member State would have been entitled to receive EISF support if the currently proposed EISF had already existed at the outbreak of the crisis in early 2020. In the subsequent section we extend this counterfactual simulation to historic data which cover, e. g., the financial crisis of 2008–10 and the sovereign debt crisis of 2011–13.

We use unemployment rates published by Eurostat to assess when and to what extent Member States would have been eligible for EISF support. In accordance with the draft law, we only consider those Member States which are either part of the Monetary Union or belong to the ERM II.^7^

It is noteworthy that unemployment rates often lag the business cycles and that there is a second, country-specific lag caused by the time span necessary to collect and compile unemployment data before the aggregate, nation-wide unemployment rate can be computed to which the double activation trigger refers.

**Fig. 1:**
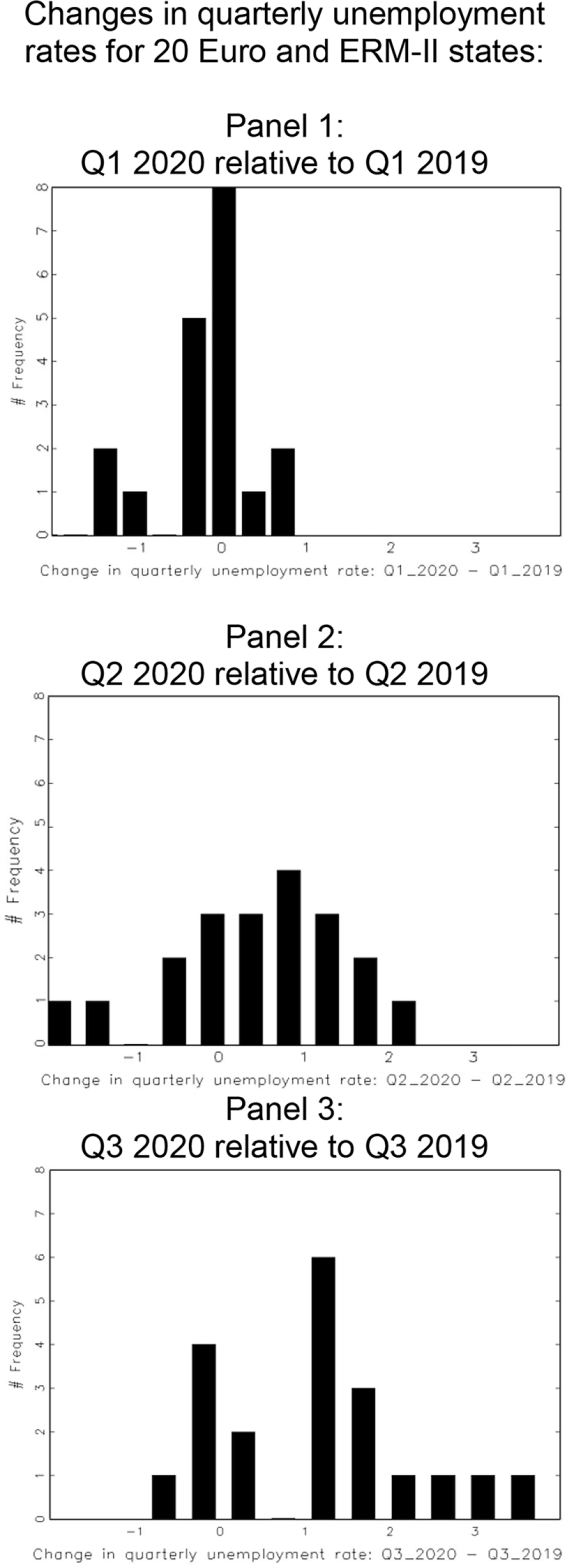
Changes in quarterly unemployment rates for 20 Euro and ERM-II states: Panel 1: Q1 2020 relative to Q1 2019. Panel 2: Q2 2020 relative to Q2 2019. Panel 3: Q3 2020 relative to Q3 2019

For instance, the Covid-19 crisis broke out in the first quarter of 2020. At the end of this quarter, i. e. by March 31, most countries had imposed a lockdown on their economies and it was perfectly clear that a major contraction was taking place. Yet, in most Eurozone countries, first quarter unemployment rates were unchanged or even lower than in the first quarter of the previous year, cf. panel 1 in Fig. 1. The unemployment toll of the crisis materialized only gradually. This can be seen by the histograms of unemployment rate changes in Fig. 1, which shift more and more into positive territory as time goes by. (Cf. panels 2 and 3 for the second and the third quarter annual changes of unemployment rates in the Eurozone.)

Second quarter unemployment rates were released by Eurostat September 8, 2020. This release was still incomplete with some countries reporting later. Hence, the Commission would have received requests for EISF support at the earliest by September 2020 and it seems unlikely that, even with speedy decisions from the side of the Commission, much EISF-funded public investment would have taken place prior to the first quarter of 2021. In other words: although rules-based and designed for enabling swift action, EISF-based macroeconomic stabilisation would not have come into effect much earlier than almost one year after a severe economic shock hit the Eurozone.

This assumes countries were eligible for EISF support already with second-quarter unemployment rates. However, in terms of unemployment, the worst was yet to come. When Eurostat released second-quarter unemployment rates, only Austria met the double activation trigger. As panel 3 of Fig. 1 shows, many countries experienced substantial unemployment increases only in quarter 3. And even then, not more than six countries qualified for EISF support: Denmark, Finland, Luxembourg, Austria, Estonia and Lithuania.

It is stunning to see that four of these six countries are among the richest of the Eurozone. Table 1 lists today’s EU Member States in descending order of their per-capita GDPs (as of 2019) along with the EISF support they would have received in response to Covid-19. Four of the seven most prosperous Member States would have received EISF support, totaling 89 billion €. Of the 13 less wealthy states, only two small countries, Estonia and Lithuania, would have been eligible (with EISF loans of 34 billion €). Thus, almost three quarters of all Covid related EISF funds would have been granted to Member States with high per-capita incomes. Except for the two Baltic states, no other EU country would have received support, with the five poorest countries (Hungary, Poland, Croatia, Romania, Bulgaria) not even being eligible.

**Table 1:** The Covid-19 Recession

Country	GDP per capita 2019 (1000 Euros)	Projected GDP growth 2020	EISF support (million Euros)
Luxembourg	102	– 0.05	11,237
Ireland	72	– 0.02	
Denmark	54	– 0.03	19,113
Netherlands	47	– 0.03	
Sweden	46	– 0.02	Not eligible
Austria	45	– 0.05	24,173
Finland	44	– 0.03	34,346
Germany	42	– 0.03	
Belgium	41	– 0.07	
France	36	– 0.07	
Italy	30	– 0.09	
Malta	27	– 0.07	
Spain	26	– 0.12	
Cyprus	25	– 0.05	
Slovenia	23	– 0.05	
Estonia	21	– 0.04	13,095
Czechia	21	– 0.04	Not eligible
Portugal	21	– 0.07	
Lithuania	17	– 0.01	20,345
Slovakia	17	– 0.04	
Greece	17	– 0.10	
Latvia	16	– 0.05	
Hungary	15	– 0.01	Not eligible
Poland	14	0.00	Not eligible
Croatia	13	– 0.09	Not eligible
Romania	12	– 0.03	Not eligible
Bulgaria	9	– 0.04	Not eligible

Source: Eurostat and own calculations

Supporting the rich and not supporting the poorest is the opposite of what common sense would expect from EU cohesion policy. In the Covid-19 crisis, EISF support would have *aggravated* income inequality in the EU rather than ameliorated it. Even in terms of stabilisation policy, the EISF would have missed its target almost completely. Column 3 of Table 1 lists the 2020 GDP growth rates as projected by the Commission. The (unweighted) average of these growth rates is  – 0.05. There are eight Member States whose GDP is projected to shrink by more than this: Belgium ( – 0.07), France ( – 0.07), Malta ( – 0.07), Portugal ( – 0.07), Italy ( – 0.09), Croatia ( – 0.09), Greece ( – 0.10), Spain ( – 0.12). None of these countries most affected by the recent crisis would have received any EISF support.

Coincidentally (or not?), some of the hardest hit countries in the Covid-19 crisis featured also prominently in the EU’s sovereign debt crisis of 2010–13. High unemployment in these countries during the sovereign debt crisis and the preceding financial crisis of 2008–09 is partially responsible for these countries not meeting the criteria of the double activation trigger. Hence, countries which suffered major crises in the past are less likely to enjoy EISF support than others.

However, it would probably be inappropriate to just do away with such long-run averages of past macroeconomic data since these come close to the notion of a country’s steady state. EU resources are intended to address major economic crises only and these are best seen as major downside deviations from the long-run equilibrium. By contrast, sudden declines in annual growth rates are not, by themselves, sufficient evidence of major crises, as such events may also occur e. g. when overheated economies experience some sort of sharp correction. It is not obvious that debt-financed interventions should take place in these cases.

Hence, it seems that both in terms of cohesion and macroeconomic stabilisation the EISF would have missed or even undermined important EU policy targets. We proceed to check if such results feature also in previous crises. For this purpose, we now check the functioning of the EISF in historic perspective.

## Historic simulation and disincentive effects

In this section, we counterfactually assume that the EISF was already in existence since inception of the Monetary Union in 1999. In accordance with the legislative proposal, we only examine Member States of the Monetary Union or those participating in the ERM II at the respective time. In 1999, this group was made up of the founding countries plus Greece and Denmark. In 2004, it was joined by Estonia, Lithuania, Slovenia and in 2005 by Latvia, Malta, Slovakia and Cyprus.

Table 2 (in the Appendix) summarizes the year and amount that each Member State would have been entitled to request EISF funds^8^ based on formula (1). We assume that, if necessary, the lending capacity of the EISF would have been increased by loans from the ESM, cf. Article 10 EISF. Essentially, the (fictitious) claims are concentrated in two periods: the years 2003–05 and the years 2008–14.

**Table 2:** Historical Simulation: Entitlements to EISF loans according to the Commission’s proposal (in billion €)

	1999	2000–2002	2003	2004	2005	2006–2007	2008	2009	2010	2011	2012	2013	2014	2015–2018
Austria	–	–	–	5	–	–	–	40	–	–	8	–	–	–
Belgium	–	–	–	–	–	–	–	–	–	–	–	9	19	–
Cyprus	–	–	–	–	0,3	–	–	4	3	0,5	0,3	0,1	–	–
Denmark	–	–	–	–	–	–	–	55	34	–	–	–	–	–
Estonia	–	–	–	–	–	–	–	5	0,5	–	–	–	–	–
Finland	–	–	–	–	–	–	–	–	–	–	–	–	–	–
France	–	–	–	–	–	–	–	–	318	–	–	–	–	–
Germany	–	–	45	140	–	–	–	–	–	–	–	–	–	–
Greece	3	–	–	–	–	–	–	–	75	14	–	–	–	–
Ireland	–	–	–	–	–	–	61	5	4	1	–	–	–	–
Italy	–	–	–	–	–	–	–	–	–	–	423	211	–	–
Latvia	–	–	–	–	–	–	–	7	1	–	–	–	–	–
Lithuania	–	–	–	–	–	–	–	9	1	–	–	–	–	–
Luxembourg	–	–	1	3	–	–	–	–	–	–	–	–	–	–
Malta	–	–	–	–	–	–	–	0.3	–	–	–	–	–	–
Netherlands	–	–	–	11	–	–	–	–	46	–	–	50	–	–
Portugal	–	–	26	19	3	–	–	8	10	–	4	–	–	–
Slovakia	–	–	–	–	–	–	–	–	21	–	–	–	–	–
Slovenia	–	–	–	–	–	–	–	10	4	1	0,5	–	–	–
Spain	–	–	–	–	–	–	–	371	30	19	7	–	–	–

Source: Own calculations

For the first period, countries with high per-capita income like Germany, Luxembourg, the Netherlands and Austria would have been granted 81 percent of the entire financial aid. Among the poorer countries only Portugal and Cyprus would have been eligible and would have received the remaining 19 percent. Despite the significantly higher risk premiums for Portugese and Cyprian government bonds, the same regressive pattern would have held for interest subsidies.

The second period was predominantly characterized by the financial crisis and the Euro crisis, in which seventeen countries would have been eligible for EISF loans—the entire Eurozone excluding Germany, Finland and Luxembourg. For the period of the financial crisis—despite its symmetrical character—EISF loans would have been surprisingly asymmetrical. Countries that were hit particularly hard (Italy, Germany or Finland) would not have been granted financial aid, whereas countries that witnessed comparatively milder contractions (like the Netherlands, France and Austria) *would have* been entitled.

During the Euro crisis (2011 – 2014), 83 percent of the entire financial aid would have gone to just one country, Italy. The staggering amount of 634 billion € in loans combined with 334 billion € in interest subsidies stand in no comparison to any other EISF funding that was granted during that period time.

**Fig. 2:**
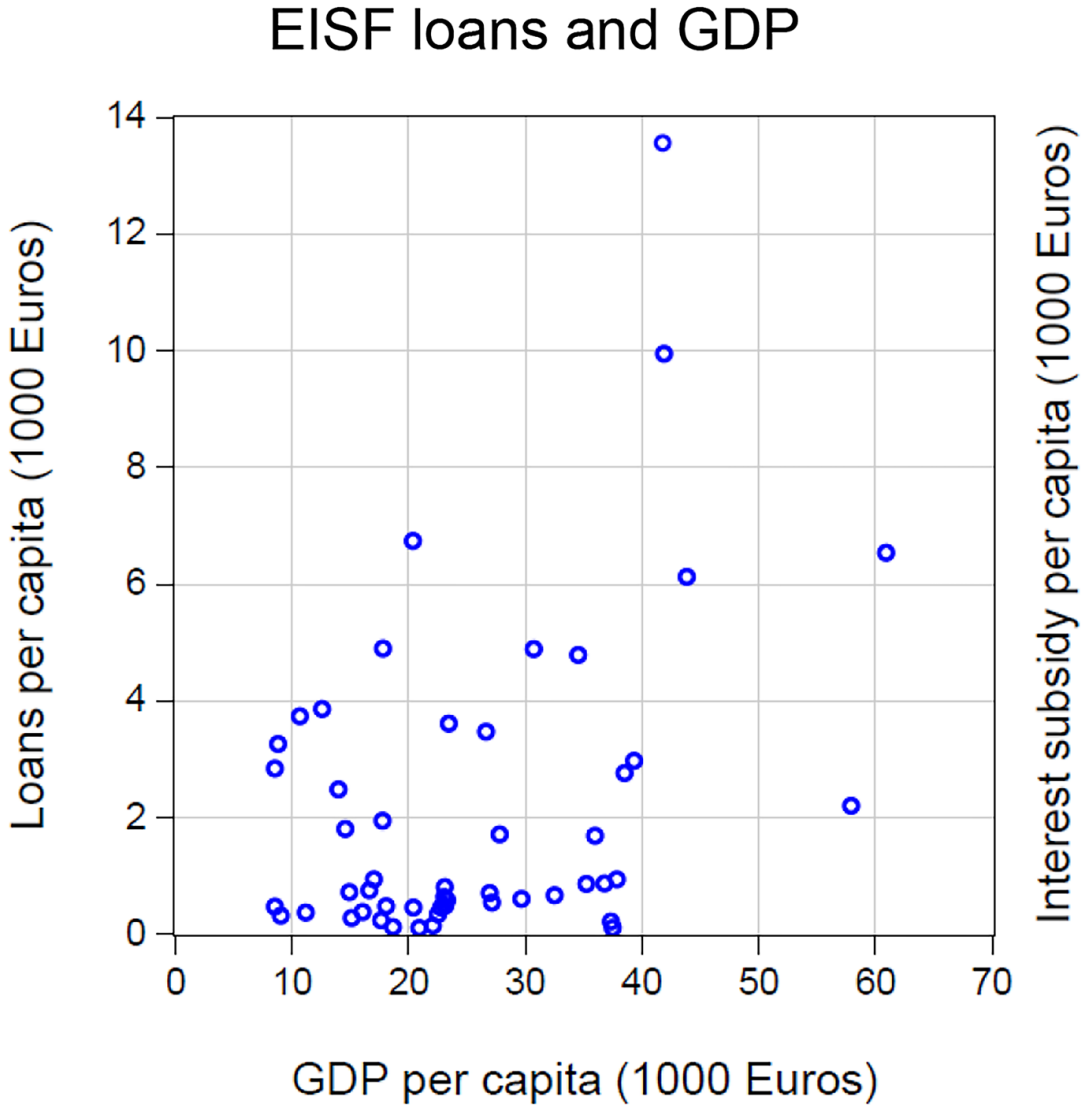
EISF loans and GDP

**Fig. 3:**
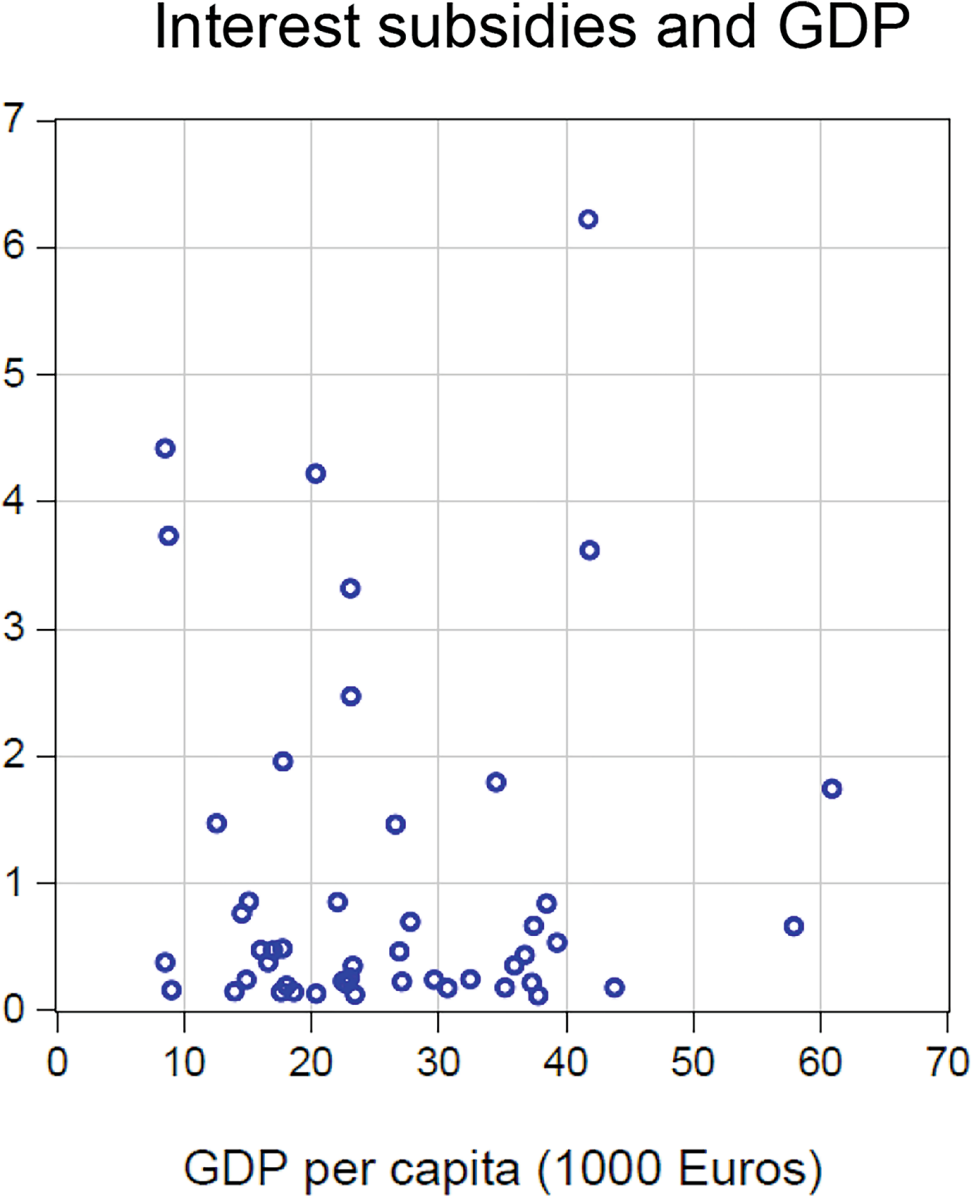
Interest subsidies and GDP

Figures 2 and 3 plot (in per-capita terms) the volume of EISF loans and interest subsidies, respectively, against each country’s per-capita income. If this financial support served cohesion, one would expect to see a negative correlation between income and financial aid. However, the converse is true. For EISF loans, the correlation is significantly positive $\left( {\rho = 0.4,\;{\text{t - stat}} = 2.8} \right)$, indicating that, if anything, the loans *increased* income inequality in the EU. The correlation between interest subsidy and income (both per capita) is almost exactly zero, so that the subsidy did not counteract the anti-cohesive effect of the EISF loans.

It is difficult to see a convincing system in the EISF’s rules-based credit allocation. In particular, it is not apparent that the EISF would be conducive to EU cohesion. The evidence points more to undermining rather than enhancing the cohesion of the Union.

As discussed in greater detail in < self-citation > , the EISF is also prone to disincentivizing timely national crisis response policies. Rapid deployment of a Member State’s own resources to prevent sharp increases in unemployment rates would forfeit its opportunity to take advantage of EISF loans and the associated interest subsidies.

For instance: For Germany, the financial crisis caused – in terms of GDP decline of -5.7 percent– the worst recession in its (postwar) economic history. But unemployment rose by not more than 0.7 percentage points. The relevant literature explains this “German employment miracle” with swift enactment of active labour market policies, where 83 billion € were spent on, e. g., short-time work schemes, cf. Möller [17], Burda and Hunt [2].

Consequently, the double activation criterion of the EISF would not have been met. Had Germany reacted more slowly when struck by the crisis, unemployment might have risen to an extent which qualified Germany for EISF support. If unemployment had risen by 1.5 (rather than 0.7) percentage points, Germany would have been eligible for roughly 300 billion € in EISF loans and 100 billion € interest subsidy over ten years.

While 100 billion € in interest subsidies is a respectable amount, we do not claim that the German government might have tolerated an increase in unemployment by 1.5 percentage points instead of 0.7. But the example demonstrates what enormous moral hazard problems smaller, financially weaker Member States would face.

In the same line of argument, note that, in the course of a crisis, unemployment may rise over several quarters. If the affected countries wanted to maximize EISF assistance, the earliest possible application date is not necessarily the optimal application date. For instance, delaying its application by just one quarter in 2011/12, Italy would have received EISF loans of 641 billion € instead of the 423 billion €—plus 365 billion € interest subsidy over ten years rather than 245 billion €.

It would be advisable to adapt the draft law such that EISF support can only be applied for at the earliest possible point in time and would otherwise lapse. However, this would not address the problem that affected Member States could feel tempted to implement their own rapid anti-crisis measures in a reduced or delayed manner to gain greater access to EU funds.

Finally, it should be noted that the EISF provisions represent a significant departure from the principle of conditionality which guided previous EU-funded assistance. Opinions may be divided on how successful the policy of conditionality has been, but the EISF gives up completely on tying lending to a commitment to structural reforms.

## Conclusions

There is wide consensus that the best economic policy response to Covid-19 is debt-financed expansionary fiscal policy. A number of discretionary measures to support such policies were rapidly designed at EU level shortly after the outbreak of the pandemic. But most of these have been slow to take effect, some not being used at all and others still awaiting to be put into force by the end of 2020.

The Commission’s draft law on a EU-wide macroeconomic stabilisation function (EISF) would replace discretionary decision making by a rules-based approach. Guaranteed by the EU budget and, if necessary, by the ESM, loans to Member States would be semi-automatically determined and made attractive by an interest subsidy financed by all Eurozone countries. If put into law, political conflicts between Member States would no longer delay the swift provision of funds to national governments.

However, a detailed analysis of this proposal casts serious doubts on the idea of a rules-based crisis response. The EISF is prone to misallocations which would be distributionally regressive, undermine Union-wide cohesion, disincentivise prompt national economic policy responses and cause negative budgetary externalities to other Eurozone countries. Moreover, the principle of conditionality, i. e. assistance provided conditional on pledges of structural reforms on the part of the recipient country, is abandoned.

Many of these weaknesses have their roots in EU primary law. The Treaties do not authorize EU institutions to conduct macroeconomic stabilisation, but reserve this as an exclusive Member State competence. To circumvent this, the Commission proposes to view macroeconomic stabilisation by its mandate for cohesion policy. But while the Treaties foresee cohesion policy to apply to the Union as a whole, the EISF proposal would provide macroeconomic stabilisation just for the enlarged Eurozone.

Without Treaty change, tying macroeconomic stabilisation to EU cohesion policy seems unavoidable. This entails the risk of grave misallocations: Since the stabilisation function must be of a cohesion policy nature, it will not necessarily be possible to use the loans optimally in terms of stabilisation policy. Cohesion support is targeted at disadvantaged regions or sectors in need of structural adjustment, while stabilisation policy is intended to have general macroeconomic effects. Cohesion policy is tailored to specific idiosyncratic and asymmetric circumstances, while stabilisation policy shall counter any kind of adverse shock which hits the whole macroeconomy. Finally, cohesion investments are designed to have medium or long-term impact, while the effects of stabilisation policy should unfold as rapidly as possible.

Anchoring macroeconomic stabilisation in this manner threatens to strip cohesion policy of its essence, as funds intended for classic cohesion policies might be diverted and flow into extraneous uses. This would have a negative impact on the EU's disadvantaged regions and sectors.

In addition, when it comes to the practical implementation, there would also be considerable doubt as to whether stabilisation support under the EISF does enhance the Union’s cohesion. Both our Covid-19 analysis and our historic simulation show that a major part of loans and interest rate subsidies would have gone to some of the wealthiest states of the Union while poorer and more severely affected countries would have received little or no support.

Additionally, poorer southern or eastern Member states, which do not belong to the Eurozone, would not receive any stabilisation support. In the event of a crisis, the cohesion of the EU would probably be reduced rather than promoted by the EISF for these countries. Moreover, each recipient state may spend the funds for example, to promote regions with high growth potential, as it is nowhere specified to support backward regions. This would undoubtedly run against the purpose of within-country cohesion.

Further problems develop as the EISF creates considerable incentive problems and may give rise to undesirable attentism in national crisis responses. This aggravates the initial effects of an adverse shock, may lead to higher risk premiums on capital markets and, therefore, have negative externalities for the countries which finance the interest subsidy.

At the EU level, it seems doubtful whether rules-based approaches to macroeconomic stabilisation are a viable alternative to discretionary decision making in the face of a crisis. Discretionary measures, on the other hand, are unsatisfactory on own account because of considerable time lags and political hurdles delaying their entry into force. We are led to the conclusion that, either way, the potential for macroeconomic stabilisation on EU level is, at best, very limited. In realistic settings well-intended measures may actually turn out to be counterproductive.

Given these problems, the best precautionary measure seems to be sufficient fiscal space on the national level. If government debt-to-GDP ratios in normal times are well below the 60% threshold enshrined in EU law, no need would arise to provide Union loans to troubled Member States.

The high degree of integration of the EU’s Common Market would ensure that expansionary fiscal policy at the national level would generate substantial spillover effects to the benefit of other Member States. Thus, a truly European crisis response may well unfold without interference by European Union institutions. Given sustainable debt levels, the key issue seems to be speedy decision making and implementation. It is quite likely that this is much easier achieved in a decentralized way by national governments – and with results more beneficial to the well-being of the Union.

## Footnotes

1 The file was apparently delayed by the election of a new parliament in May 2019 and the outbreak of the pandemic in early 2020.

2 COM(2018) 387 final.

3 We will not deal with the BICC in this paper as the proposal is still quite sketchy and lacks a legal text.

4 Eligible are Member States which are neither subject to a macroeconomic adjustment programme nor benefit from financial assistance by the European Stability Mechanism (ESM). Moreover, they must not have been reproved by the European Council for failure to take effective measures against macroeconomic or budgetary imbalances in the two years prior to requesting EISF support.

5 The Commission justifies the values as follows: $\alpha$ “is determined such that with hindsight of the recent crisis, all the EISF support could have been provided to the Member States concerned, had the mechanism been in place.” $\beta$ is „determined such that for a shock that increases unemployment by more than 2.5 percentage points, the maximum support is made available to the Member State concerned. “ Cf. Recitals 23 and 24 of the Commission proposal.

6 COM(2018) 375 final.

7 For ease of language, „Eurozone “ will refer to the Monetary Union plus ERM II in the sequel.

8 Unemployment rates are not available for all Member States with a lead time of 60 quarters prior to a possible entitlement year. This is especially true for the reunited Germany and the former socialist states. In these cases, we have formed averages over shorter periods of time, but these averages often still cover ten years or more.
